# Supplementary material for: A systematic review of statistical methodology used to evaluate progression of chronic kidney disease using electronic healthcare records
Source: PLoS One. 2022 Jul 29;17(7):e0264167. doi: 10.1371/journal.pone.0264167 (PMC9337679; doi:10.1371/journal.pone.0264167)
Supplement: S2 File — (DOCX) [file pone.0264167.s002.docx]

**Supporting Information S2. MEDLINE database search strategy**

| # | Terms |
| --- | --- |
| 1 | (CKD or CKF or CRD or CRF or “chronic kidney disease” or “kidney disease” or “renal disease” or “kidney failure” of “renal failure”).mp. |
| 2 | exp Renal Insufficiency, Chronic/ |
| 3 | 1 or 2 |
| 4 | (“kidney function” or “renal function” or GFR or eGFR or mGFR or creatinine or “cystatin C” or “iohexol clearance” or MDRD or “CKD-EPI”).mp |
| 5 | exp Glomerular Filtration Rate/ or exp Creatinine/ or exp Cystatin C/ |
| 6 | 4 or 5 |
| 7 | ((electronic or computer* or anonymi#ed) adj3 (health* or medical or patient* or GP* or “general practictioner” or “primary care” or “hospital” or “secondary care” or observational or routine*) adj3 (record* or data*)).mp |
| 8 | ((“primary care” or GP or practice* or “secondary care” or link* or hospital* or clinic* or centre or center) adj3 (data* or record*)).mp |
| 9 | (EHR or CPR or EMR or EPR or AMR).mp |
| 10 | exp Electronic Health Records/ or exp Medical Record Linkage/ or exp Medical Records Systems, Computerized/ |
| 11 | 7 or 8 or 9 or 10 |
| 12 | (chang* or declin* or progress* or longitudinal* or trajectory* or slope* or deteriorate* or loss*).mp |
| 13 | exp Longitudinal Studies/ |
| 14 | 12 or 13 |
| 15 | ((chang* or declin* or progress* or longitudinal* or trajectory* or slope* or deteriorate* or loss*) adj3 (“kidney function” or “renal function” or GFR or eGFR or mGFR or creatinine or “cystatin C” or “iohexol clearance” or MDRD or “CKD-EPI”)).mp |
| 16 | 5 and 13 |
| 17 | 15 or 16 |
| 18 | 3 and 11 and 17 |
| 19 | exp clinical trial/ or exp case reports/ or (“clinical trial” or “randomi#ed trial” or RCT* or “case report*”).m_titl. |
| 20 | (“dialysis patient*” or “transplant patient*”).m_titl. |
| 21 | 19 or 20 |
| 22 | 18 not 21 |
