# Supplementary material for: A systematic review of statistical methodology used to evaluate progression of chronic kidney disease using electronic healthcare records
Source: PLoS One. 2022 Jul 29;17(7):e0264167. doi: 10.1371/journal.pone.0264167 (PMC9337679; doi:10.1371/journal.pone.0264167)
Supplement: S3 File — (DOCX) [file pone.0264167.s003.docx]

**Supporting Information S3. List of reviewed studies** *(article numbers match references in manuscript [for articles 11-62] and follow from manuscript reference numbers for articles not referenced in manuscript [articles 69-96])*

11. Chase HS, Hirsch JS, Mohan S, Rao MK, Radhakrishnan J. Presence of early CKD-related metabolic complications predict progression of stage 3 CKD: a case-controlled study. BMC Nephrology. 2014;15:187.

12. Wang Y, Zhao L, Zhang J, Wu Y, Zhang R, Li H, et al. Implications of a Family History of Diabetes and Rapid eGFR Decline in Patients With Type 2 Diabetes and Biopsy-Proven Diabetic Kidney Disease. Frontiers in Endocrinology. 2019;10 (no pagination).

13. Abdelhafiz AH, Tan E, Levett C, Minchin J, Nahas ME. Natural history and predictors of faster glomerular filtration rate decline in a referred population of older patients with type 2 diabetes mellitus. Hospital practice (1995) Hospital practice. 2012;40(4):49-55.

14. Eriksen BO, Ingebretsen OC. The progression of chronic kidney disease: a 10-year population-based study of the effects of gender and age. Kidney International. 2006;69(2):375-82.

15. Jalal K, Anand EJ, Venuto R, Eberle J, Arora P. Can billing codes accurately identify rapidly progressing stage 3 and stage 4 chronic kidney disease patients: a diagnostic test study. Bmc Nephrology. 2019;20.

16. Cabrera CS, Lee AS, Olsson M, Schnecke V, Westman K, Lind M, et al. Impact of CKD Progression on Cardiovascular Disease Risk in a Contemporary UK Cohort of Individuals With Diabetes. Kidney International Reports. 2020;5(10):1651-60.

17. Eriksen BO, Tomtum J, Ingebretsen OC. Predictors of declining glomerular filtration rate in a population-based chronic kidney disease cohort. Nephron. 2010;115(1):c41-50.

18. Annor FB, Masyn KE, Okosun IS, Roblin DW, Goodman M. Psychosocial stress and changes in estimated glomerular filtration rate among adults with diabetes mellitus. Kidney Research and Clinical Practice. 2015;34(3):146-53.

19. Diggle PJ, Sousa I, Asar O. Real-time monitoring of progression towards renal failure in primary care patients. Biostatistics. 2015;16(3):522-36.

20. Butt AA, Ren Y, Puenpatom A, Arduino JM, Kumar R, Abou-Samra AB. Effectiveness, treatment completion and safety of sofosbuvir/ledipasvir and paritaprevir/ritonavir/ombitasvir + dasabuvir in patients with chronic kidney disease: an ERCHIVES study. Alimentary Pharmacology & Therapeutics. 2018;48(1):35-43.

21. Singh A, Nadkarni G, Gottesman O, Ellis SB, Bottinger EP, Guttag JV. Incorporating temporal EHR data in predictive models for risk stratification of renal function deterioration. Journal of Biomedical Informatics. 2015;53:220-8.

22. Evans RDR, Cargill T, Goodchild G, Oliveira B, Rodriguez-Justo M, Pepper R, et al. Clinical Manifestations and Long-term Outcomes of IgG4-Related Kidney and Retroperitoneal Involvement in a United Kingdom IgG4-Related Disease Cohort. Kidney International Reports. 2019;4(1):48-58.25. Vejakama P, Ingsathit A, Attia J, Thakkinstian A. Epidemiological Study of Chronic Kidney Disease Progression: A Large-Scale Population-Based Cohort Study. Medicine. 2015;94(4).

23. Jackevicius CA, Lu LY, Ghaznavi Z, Warner AL. Bleeding Risk of Direct Oral Anticoagulants in Patients With Heart Failure And Atrial Fibrillation. Circulation-Cardiovascular Quality and Outcomes. 2021;14(2):155-68.

24. Lai YJ, Lin YC, Peng CC, Chen KC, Chuang MT, Wu MS, et al. Effect of weight loss on the estimated glomerular filtration rates of obese patients at risk of chronic kidney disease: the RIGOR-TMU study. Journal of Cachexia, Sarcopenia and Muscle. 2019;10(4):756-66.

25. Vejakama P, Ingsathit A, Attia J, Thakkinstian A. Epidemiological Study of Chronic Kidney Disease Progression: A Large-Scale Population-Based Cohort Study. Medicine. 2015;94(4).

26. Posch F, Ay C, Stoger H, Kreutz R, Beyer-Westendorf J. Exposure to vitamin k antagonists and kidney function decline in patients with atrial fibrillation and chronic kidney disease. Research and Practice in Thrombosis and Haemostasis. 2019;3(2):207-16.

27. Hsu TW, Hsu CN, Wang SW, Huang CC, Li LC. Comparison of the effects of denosumab and alendronate on cardiovascular and renal outcomes in osteoporotic patients. Journal of Clinical Medicine. 2019;8(7).

28. Inaguma D, Kitagawa A, Yanagiya R, Koseki A, Iwamori T, Kudo M, et al. Increasing tendency of urine protein is a risk factor for rapid eGFR decline in patients with CKD: A machine learning-based prediction model by using a big database. PLoS ONE [Electronic Resource]. 2020;15(9):e0239262.

29. Peng YL, Tain YL, Lee CT, Yang YH, Huang YB, Wen YH, et al. Comparison of uric acid reduction and renal outcomes of febuxostat vs allopurinol in patients with chronic kidney disease. Scientific Reports. 2020;10(1):10734.

30. Yao X, Tangri N, Gersh BJ, Sangaralingham LR, Shah ND, Nath KA, et al. Renal Outcomes in Anticoagulated Patients With Atrial Fibrillation. Journal of the American College of Cardiology. 2017;70(21):2621-32.36. Lv L, Chang DY, Li ZY, Chen M, Hu Z, Zhao MH. Persistent hematuria in patients with antineutrophil cytoplasmic antibody-associated vasculitis during clinical remission: chronic glomerular lesion or low-grade active renal vasculitis? BMC Nephrology. 2017;18(1):354.

31. Lamacchia O, Viazzi F, Fioretto P, Mirijello A, Giorda C, Ceriello A, et al. Normoalbuminuric kidney impairment in patients with T1DM: Insights from annals initiative. Diabetology and Metabolic Syndrome. 2018;10(1).

32. Viazzi F, Greco E, Ceriello A, Fioretto P, Giorda C, Guida P, et al. Apparent treatment resistant hypertension, blood pressure control and the progression of chronic kidney disease in patients with type 2 diabetes. Kidney and Blood Pressure Research. 2018;43(2):422-38.

33. Rej S, Herrmann N, Gruneir A, McArthur E, Jeyakumar N, Muanda FT, et al. Association of Lithium Use and a Higher Serum Concentration of Lithium With the Risk of Declining Renal Function in Older Adults: A Population-Based Cohort Study. The Journal of clinical psychiatry. 2020;81(5).

34. Yoo H, Park I, Kim DJ, Lee S. Effects of sarpogrelate on microvascular complications with type 2 diabetes. International Journal of Clinical Pharmacy. 2019.

35. Tangri N, Reaven NL, Funk SE, Ferguson TW, Collister D, Mathur V. Metabolic acidosis is associated with increased risk of adverse kidney outcomes and mortality in patients with non-dialysis dependent chronic kidney disease: an observational cohort study. BMC Nephrology. 2021;22(1).

36. Lv L, Chang DY, Li ZY, Chen M, Hu Z, Zhao MH. Persistent hematuria in patients with antineutrophil cytoplasmic antibody-associated vasculitis during clinical remission: chronic glomerular lesion or low-grade active renal vasculitis? BMC Nephrology. 2017;18(1):354.

37. Li XM, Rui HC, Liang DD, Xu F, Liang SS, Zhu XD, et al. Clinicopathological characteristics and outcomes of light chain deposition disease: an analysis of 48 patients in a single Chinese center. Annals of Hematology. 2016;95(6):901-9.

38. Gallant JE, Parish MA, Keruly JC, Moore RD. Changes in renal function associated with tenofovir disoproxil fumarate treatment, compared with nucleoside reverse-transcriptase inhibitor treatment. Clinical Infectious Diseases. 2005;40(8):1194-8.

39. Herget-Rosenthal S, Dehnen D, Kribben A, Quellmann T. Progressive chronic kidney disease in primary care: modifiable risk factors and predictive model. Preventive Medicine. 2013;57(4):357-62.

40. Morales-Alvarez MC, Garcia-Dolagaray G, Millan-Fierro A, Rosas SE. Renal Function Decline in Latinos With Type 2 Diabetes. Kidney International Reports. 2019;4(9):1230-4.

41. Nderitu P, Doos L, Strauss VY, Lambie M, Davies SJ, Kadam UT. Analgesia dose prescribing and estimated glomerular filtration rate decline: a general practice database linkage cohort study. BMJ Open. 2014;4(8):e005581.

42. Koraishy FM, Hooks-Anderson D, Salas J, Scherrer JF. Rate of renal function decline, race and referral to nephrology in a large cohort of primary care patients. Family Practice. 2017;34(4):416-22.

43. Johnson F, Phillips D, Talabani B, Wonnacott A, Meran S, Phillips AO. The impact of acute kidney injury in diabetes mellitus. Nephrology. 2016;21(6):506-11.44. Chakera A, MacEwen C, Bellur SS, Chompuk LO, Lunn D, Roberts ISD. Prognostic value of endocapillary hypercellularity in IgA nephropathy patients with no immunosuppression. Journal of Nephrology. 2016;29(3):367-75.

44. Chakera A, MacEwen C, Bellur SS, Chompuk LO, Lunn D, Roberts ISD. Prognostic value of endocapillary hypercellularity in IgA nephropathy patients with no immunosuppression. Journal of Nephrology. 2016;29(3):367-75.

45. Chen H, Liu C, Fu C, Zhang H, Yang H, Wang P, et al. Combined application of eGFR and albuminuria for the precise diagnosis of stage 2 and 3a CKD in the elderly. Journal of Nephrology. 2014;27(3):289-97.

46. Perotte A, Ranganath R, Hirsch JS, Blei D, Elhadad N. Risk prediction for chronic kidney disease progression using heterogeneous electronic health record data and time series analysis. Journal of the American Medical Informatics Association. 2015;22(4):872-80.

47. Cummings DM, Larsen LC, Doherty L, Lea CS, Holbert D. Glycemic Control Patterns and Kidney Disease Progression among Primary Care Patients with Diabetes Mellitus. Journal of the American Board of Family Medicine. 2011;24(4):391-8.

48. Horne L, Ashfaq A, MacLachlan S, Sinsakul M, Qin L, LoCasale R, et al. Epidemiology and health outcomes associated with hyperkalemia in a primary care setting in England. BMC Nephrology. 2019;20(1):85.

49. Robinson DE, Ali MS, Pallares N, Tebe C, Elhussein L, Abrahamsen B, et al. Safety of Oral Bisphosphonates in Moderate-to-Severe Chronic Kidney Disease: A Binational Cohort Analysis. Journal of Bone and Mineral Research. 2021;36(5):820-32.

50. Nichols GA, Deruaz-Luyet A, Brodovicz KG, Kimes TM, Rosales AG, Hauske SJ. Kidney disease progression and all-cause mortality across estimated glomerular filtration rate and albuminuria categories among patients with vs. Without type 2 diabetes. BMC Nephrology. 2020;21(1).

51. Yanagawa T, Koyano K, Azuma K. Retrospective study of factors associated with progression and remission/regression of diabetic kidney disease-hypomagnesemia was associated with progression and elevated serum alanine aminotransferase levels were associated with remission or regression. Diabetology International. 2021;12(3):268-76.

52. Vesga JI, Cepeda E, Pardo CE, Paez S, Sanchez R, Sanabria RM. Chronic kidney disease progression and transition probabilities in a large preventive cohort in colombia. International Journal of Nephrology. 2021;2021 (no pagination).

53. Oetjens M, Bush WS, Birdwell KA, Dilks HH, Bowton EA, Denny JC, et al. Utilization of an EMR-biorepository to identify the genetic predictors of calcineurin-inhibitor toxicity in heart transplant recipients. Pacific Symposium on Biocomputing. 2014:253-64.

54. Neuen BL, Weldegiorgis M, Herrington WG, Ohkuma T, Smith M, Woodward M. Changes in GFR and Albuminuria in Routine Clinical Practice and the Risk of Kidney Disease Progression. American Journal of Kidney Diseases. 2021.

55. Weldegiorgis M, Smith M, Herrington WG, Bankhead C, Woodward M. Socioeconomic disadvantage and the risk of advanced chronic kidney disease: results from a cohort study with 1.4 million participants. Nephrology Dialysis Transplantation. 2020;35(9):1562-70.

56. Niu SF, Wu CK, Chuang NC, Yang YB, Chang TH. Early Chronic Kidney Disease Care Programme delays kidney function deterioration in patients with stage I-IIIa chronic kidney disease: an observational cohort study in Taiwan. Bmj Open. 2021;11(1).

57. O'Riordan A, Dutt N, Cairns H, Rela M, O'Grady JG, Heaton N, et al. Renal biopsy in liver transplant recipients. Nephrology Dialysis Transplantation. 2009;24(7):2276-82.

58. Tsai CW, Lin SY, Kuo CC, Huang CC. Serum Uric Acid and Progression of Kidney Disease: A Longitudinal Analysis and Mini-Review. PLoS ONE [Electronic Resource]. 2017;12(1):e0170393.

59. Leither MD, Murphy DP, Bicknese L, Reule S, Vock DM, Ishani A, et al. The impact of outpatient acute kidney injury on mortality and chronic kidney disease: a retrospective cohort study. Nephrology Dialysis Transplantation. 2019;34(3):493-501.

60. Liu D, You J, Liu Y, Tang X, Tan X, Xia M, et al. Serum immunoglobulin G provides early risk prediction in immunoglobulin A nephropathy. International Immunopharmacology. 2019;66:13-8.

61. Rincon-Choles H, Jolly SE, Arrigain S, Konig V, Schold JD, Nakhoul G, et al. Impact of Uric Acid Levels on Kidney Disease Progression. American Journal of Nephrology. 2017;46(4):315-22.69. Joss N, Paterson KR, Deighan CJ, Simpson K, Boulton-Jones JM. Diabetic nephropathy: how effective is treatment in clinical practice? Qjm. 2002;95(1):41-9.

62. VanWagner LB, Montag S, Zhao L, Allen NB, Lloyd-Jones DM, Das A, et al. Cardiovascular Disease Outcomes Related to Early Stage Renal Impairment After Liver Transplantation. Transplantation. 2018;102(7):1096-107.

69. Joss N, Paterson KR, Deighan CJ, Simpson K, Boulton-Jones JM. Diabetic nephropathy: how effective is treatment in clinical practice? Qjm. 2002;95(1):41-9.

70. Dean BB, Dylan M, Gano A, Knight K, Ofman JJ, Levine BS. Erythropoiesis-stimulating protein therapy and the decline of renal function: a retrospective analysis of patients with chronic kidney disease. Current Medical Research and Opinion. 2005;21(7):981-7.

71. Jones C, Roderick P, Harris S, Rogerson M. An evaluation of a shared primary and secondary care nephrology service for managing patients with moderate to advanced CKD. American Journal of Kidney Diseases. 2006;47(1):103-14.

72. Chen SC, Chang JM, Chou MC, Lin MY, Chen JH, Sun JH, et al. Slowing renal function decline in chronic kidney disease patients after nephrology referral. Nephrology. 2008;13(8):730-6.

73. Boudville N, Kemp A, Moody H, Fassett RG, Pedagogos E, Nelson C, et al. Factors associated with chronic kidney disease progression in Australian nephrology practices. Nephron - Clinical Practice. 2012;121(1-2):c36-c41.

74. Dreyer G, Hull S, Mathur R, Chesser A, Yaqoob MM. Progression of chronic kidney disease in a multi-ethnic community cohort of patients with diabetes mellitus. Diabetic Medicine. 2013;30(8):956-63.

75. Malgor RD, Oderich GS, Vrtiska TJ, Kalra M, Duncan AA, Gloviczki P, et al. A case-control study of intentional occlusion of accessory renal arteries during endovascular aortic aneurysm repair. Journal of Vascular Surgery. 2013;58(6):1467-75.

76. Brosnan EM, Weickhardt AJ, Lu X, Maxon DA, Baron AE, Chonchol M, et al. Drug-induced reduction in estimated glomerular filtration rate in patients with ALK-positive non-small cell lung cancer treated with the ALK inhibitor crizotinib. Cancer. 2014;120(5):664-74.

77. Kose E, An T, Kikkawa A, Matsumoto Y, Hayashi H. Effects on serum uric acid by difference of the renal protective effects with atorvastatin and rosuvastatin in chronic kidney disease patients. Biological & Pharmaceutical Bulletin. 2014;37(2):226-31.

78. Cid Ruzafa J, Paczkowski R, Boye KS, Di Tanna GL, Sheetz MJ, Donaldson R, et al. Estimated glomerular filtration rate progression in UK primary care patients with type 2 diabetes and diabetic kidney disease: a retrospective cohort study. International Journal of Clinical Practice. 2015;69(8):871-82.

79. Kaga M, Utsumi T, Tanaka T, Kono T, Nagano H, Kawamura K, et al. Risk of New-Onset Dyslipidemia After Laparoscopic Adrenalectomy in Patients with Primary Aldosteronism. World Journal of Surgery. 2015;39(12):2935-40.

80. Lai CL, Chou HW, Chan KA, Lai MS. Effects of atorvastatin and rosuvastatin on renal function in patients with type 2 diabetes mellitus. American Journal of Cardiology. 2015;115(5):619-24.

81. Yun WS. Long-Term Follow-Up Results of Acute Renal Embolism after Anticoagulation Therapy. Annals of Vascular Surgery. 2015;29(3).

82. Kim YG, Byun J, Yoon D, Jeon JY, Han SJ, Kim DJ, et al. Renal Protective Effect of DPP-4 Inhibitors in Type 2 Diabetes Mellitus Patients: A Cohort Study. Journal of Diabetes Research. 2016.

83. Mirajkar N, Bellary S, Ahmed M, Singhal R, Daskalakis M, Tahrani AA. The impact of bariatric surgery on estimated glomerular filtration rate in patients with type 2 diabetes: a retrospective cohort study. Surgery for Obesity & Related Diseases. 2016;12(10):1883-9.

84. Nishida Y, Takahashi Y, Tezuka K, Takeuchi S, Nakayama T, Asai S. Comparative Effect of Calcium Channel Blockers on Glomerular Function in Hypertensive Patients with Diabetes Mellitus. Drugs in R&D. 2017;17(3):403-12.

85. Beyer-Westendorf J, Kreutz R, Posch F, Ay C. The CHA2DS2VASc score strongly correlates with glomerular filtration rate and predicts renal function decline over time in elderly patients with atrial fibrillation and chronic kidney disease. International Journal of Cardiology. 2018;253:71-7.

86. Kim WJ, Song JS, Choi ST. The role of a "treat-to-target" approach in the long-term renal outcomes of patients with gout. Journal of Clinical Medicine. 2019;8(7).

87. O'Neill RA, Gallagher P, Douglas T, Little JA, Maxwell AP, Silvestri G, et al. Evaluation of long-term intravitreal anti-vascular endothelial growth factor injections on renal function in patients with and without diabetic kidney disease. BMC Nephrology. 2019;20(1).

88. Park JM, Yang SW, Shin JH, Na YG, Song KH, Lim JS. Oncological and Functional Outcomes of Laparoscopic Radiofrequency Ablation and Partial Nephrectomy for T1a Renal Masses: A Retrospective Single-center 60 Month Follow-up Cohort Study. Urology Journal. 2019;16(1):44-9.

89. Posch F, Ay C, Stoger H, Kreutz R, Beyer-Westendorf J. Longitudinal kidney function trajectories predict major bleeding, hospitalization and death in patients with atrial fibrillation and chronic kidney disease. International Journal of Cardiology. 2019;282:47-52.

90. Spanopoulos D, Okhai H, Zaccardi F, Tebboth A, Barrett B, Busse M, et al. Temporal variation of renal function in people with type 2 diabetes mellitus: A retrospective UK clinical practice research datalink cohort study. Diabetes, Obesity and Metabolism. 2019;21(8):1817-23.

91. Zhao J, Gu S, McDermaid A. Predicting outcomes of chronic kidney disease from EMR data based on Random Forest Regression. Mathematical Biosciences. 2019;310:24-30.

92. Lee JS, Oh JS, Kim YG, Lee CK, Yoo B, Hong S. Recovery of renal function in patients with lupus nephritis and reduced renal function: the beneficial effect of hydroxychloroquine. Lupus. 2020;29(1):52-7.

93. Nakamura A, Miyoshi H, Kameda H, Yamashita K, Kurihara Y. Impact of sodium-glucose cotransporter 2 inhibitors on renal function in participants with type 2 diabetes and chronic kidney disease with normoalbuminuria. Diabetology and Metabolic Syndrome. 2020;12(1).

94. Sise ME, Chute DF, Oppong Y, Davis MI, Long JD, Silva ST, et al. Direct-acting antiviral therapy slows kidney function decline in patients with Hepatitis C virus infection and chronic kidney disease. Kidney International. 2020;97(1):193-201.

95. Cleary F, Prieto-Merino D, Hull S, Caplin B, Nitsch D. Feasibility of evaluation of the natural history of kidney disease in the general population using electronic healthcare records. Clinical Kidney Journal. 2020;14(6):1603-9.

96. Faraj KS, Mi L, Eversman S, Singh R, DeLucia NM, Blodgett G, et al. The effect of urinary diversion on long-term kidney function after cystectomy. Urologic Oncology: Seminars and Original Investigations. 2020;38(10):796.e15-.e21.
