# Supplementary material for: A systematic review of statistical methodology used to evaluate progression of chronic kidney disease using electronic healthcare records
Source: PLoS One. 2022 Jul 29;17(7):e0264167. doi: 10.1371/journal.pone.0264167 (PMC9337679; doi:10.1371/journal.pone.0264167)
Supplement: S1 Table — (DOCX) [file pone.0264167.s004.docx]

**Table S1. Summary of study populations, where unclear if EHRs used (N = 10)**

| **Study population characteristics** | **N (%)** |
| --- | --- |
| Primary decade of follow up  2010-2019  2000-2009  1990-1999  Not available | 3 (30.0%)  6 (60.0%)  0  1 (10.0%) |
| Country  **Europe**  UK  **North America**  USA  **Asia**  South Korea  China  Japan  **Oceania** | **1 (10.0%)**  1 (10.0%)  **1 (10.0%)**  1 (10.0%)  **8 (80.0%)**  2 (20.0%)  4 (40.0%)  2 (20.0%)  **1 (1.5%)** |
| Mean age^a^  Median (IQR)  30-49  50-59  60-69  70-80  Not stated | 59 (53, 68)  1 (10.0%)  5 (50.0%)  2 (20.0%)  2 (20.0%)  0 |
| Percent male  Median (IQR)  ≤ 34%  35-44%  45-54%  55-64%  ≥ 65% | 57% (48%, 79%)  0  2 (20.0%)  3 (30.0%)  1 (10.0%)  4 (40.0%) |
| Main morbidity /reason for inclusion  Diabetes  CKD  IgA nephropathy  Other | 1 (10.0%)  1 (10.0%)  1 (10.0%)  7 (70.0%) |
| Data source / clinical setting  Multiple care settings  Outpatient  Diabetes clinic  Hospital  Tertiary care  Not stated | 1 (10.0%)  1 (10.0%)  1 (9.2%)  6 (60.0%)  1 (10.0%)  1 (10.0%) |

^a^If mean age unavailable, median used.
