# Supplementary material for: A systematic review of statistical methodology used to evaluate progression of chronic kidney disease using electronic healthcare records
Source: PLoS One. 2022 Jul 29;17(7):e0264167. doi: 10.1371/journal.pone.0264167 (PMC9337679; doi:10.1371/journal.pone.0264167)
Supplement: S2 Table — (DOCX) [file pone.0264167.s005.docx]

**Table S2. Study methodology, where unclear if EHRs used (N=10)**

| **Study methodology features** | **N (%)** |
| --- | --- |
| Date of publication  2015-2020  2010-2014 | 7 (70.0%)  3 (30.0%) |
| Study design  Retrospective cohort study | 10 (100%) |
| Research aims  Risk factor identification / casual inference  Estimation of incidence/prevalence  Descriptive characterisation of changes in renal function | 8 (80.0%)  1 (10.0%)  1 (10.0%) |
| Sample size  Median (IQR)  ≤ 99  100 – 499 | 101 (41, 247)  5 (50.0%)  5 (50.0%) |
| Measure of renal function  **eGFR**  MDRD  CKD-EPI  Japanese formula  Not specified  **Serum creatinine** | **8 (80.0%)**  3 (37.5%)  1 (12.5%)  1 (12.5%)  3 (37.5%)  **2 (20.0%)** |
| Measure of change in renal function over time  **eGFR**  Rate of change between measures  Rate of percentage change, not clearly defined  Raw absolute change from baseline  Raw percent change from baseline  Binary progression (changes/threshold combination)    **Serum creatinine**  Raw absolute change from baseline  Binary progression to threshold serum creatinine | **8 (80.0%)**  1 (10.0%)  1 (10.0%)  3 (30.0%)  2 (20.0%)  1 (10.0%)  **2 (20.0%)**  1 (10.0%)  1 (10.0%) |
| Change in renal function as outcome or exposure  Outcome  Exposure | 100 (100%)  0 |
| Duration of follow up for renal function changes  1 – 4.9 years  5 – 9.9 years | 8 (80.0%)  2 (20.0%) |
| Minimum number of renal function measures for inclusion  2  3  Not stated | 4 (40.0%)  1 (10.0%)  5 (50.0%) |
| Percentage of target population used in analysis  50% - 75%  75% - 90%  90% - 95%  >95%  Not available | 2 (20.0%)  1 (10.0%)  1 (10.0%)  1 (10.0%)  5 (50.0%) |
| Percentage of study population lost to follow up  Not available or not relevant | 10 (100%) |
| Statistical tools used^a^  Descriptive results only  Simple frequentist methods  ANCOVA  Generalised linear models (GLMs)  Cox proportional hazards regression | 2 (20.0%)  3 (30.0%)  1 (10.0%)  1 (10.0%)  3 (30.0%) |
| Statistical model used^a^  **Risk factor identification / casual inference**  Difference in means t-test  Mean difference paired t-test  ANOVA  ANCOVA  Logistic regression  Cox proportional hazards regression  **Estimation of incidence/prevalence**  Crude estimation | **N = 8**  1 (10.0%)  1 (10.0%)  1 (10.0%)  1 (10.0%)  1 (10.0%)  3 (30.0%)  **N = 1**  1 (10.0%) |

^a^Multiple items possible for a single study but focus only on main analysis of CKD progression
