# Supplementary material for: A systematic review of statistical methodology used to evaluate progression of chronic kidney disease using electronic healthcare records
Source: PLoS One. 2022 Jul 29;17(7):e0264167. doi: 10.1371/journal.pone.0264167 (PMC9337679; doi:10.1371/journal.pone.0264167)
Supplement: S3 Table — (DOCX) [file pone.0264167.s006.docx]

**Table S3. Critique of handling of data quality and methodological challenges, where unclear if EHRs used (N = 10)**

| **Handling of data quality and methodological challenges** | **N (%)** |
| --- | --- |
| Handling of sample representativeness of target population used for analysis  Not mentioned  Mentioned care pathway and inclusion criteria, but not sample completeness  Mentioned sample completeness, but not implications  Partially acknowledged implications of sample completeness  Fully acknowledged implications of sample completeness  Tackled methodologically  Methods of handling  None | 3 (30.0%)  1 (10.0%)  0  5 (50.0%)  1 (10.0%)  0  10 (100%) |
| Handling of informative drop-outs/censoring  Not mentioned  Mentioned care pathway follow up, but not losses to follow up (inc. death)  Mentioned losses to follow up, but not implications  Partially acknowledged implications of losses to follow up  Fully Acknowledged implications of losses to follow up  Tackled methodologically  Methods of handling  None | 8 (80.0%)  0  2 (20.0%)  0  0  0  10 (100%) |
| Handling of missing longitudinal data  Not mentioned  Mentioned care pathway follow up, but not data completeness  Mentioned data completeness, but not implications  Partially acknowledged implications of data completeness  Fully acknowledged implications of data completeness  Tackled methodologically  Methods of handling  None | 10 (100%)  0  0  0  0  0  10 (100%) |
| Handling of missing covariate data  Not mentioned  Mentioned data completeness, but not implications  Partially acknowledged implications of data completeness  Fully acknowledged implications of data completeness  Tackled methodologically or not an issue  Methods of handling  None | 6 (60.0%)  0  1 (10.0%)  0  3 (30.0%)  10 (100%) |
| Distributional checks/issues  Not mentioned  Mentioned or partially addressed  Fully Acknowledged  Tackled  Methods of handling  None | 10 (100%)  0  0  0  10 (100%) |
| Handling of within-patient correlation / variability in kidney function over time  Not mentioned  Mentioned or partially addressed  Fully Acknowledged  Tackled  Methods of handling  None  Outcome likely to identify real change | 7 (70.0%)  3 (30.0%)  0  0  8 (80.0%)  2 (20.0%) |
| Handling of population heterogeneity  Not mentioned  Mentioned or partially addressed  Fully Acknowledged  Tackled  Method of handling  None  Adjustment for covariates  Stratified or separate/subgroup analysis  ANOVA/ANCOVA | 1 (10.0%)  5 (50.0%)  1 (10.0%)  3 (30.0%)  3 (30.0%)  1 (10.0%)  5 (50.0%)  1 (10.0%) |
| Handling of confounding (risk factor / causal inference analyses only)  Not mentioned  Mentioned or partially addressed  Fully Acknowledged  Tackled  Methods  None  Adjustment for baseline confounders | N = 8  4 (50.0%)  2 (25.0%)  0  2 (25.0%)  4 (50.0%)  4 (50.0%) |
