# Supplementary material for: A systematic review of statistical methodology used to evaluate progression of chronic kidney disease using electronic healthcare records
Source: PLoS One. 2022 Jul 29;17(7):e0264167. doi: 10.1371/journal.pone.0264167 (PMC9337679; doi:10.1371/journal.pone.0264167)
Supplement: S4 Table — (DOCX) [file pone.0264167.s007.docx]

**S4 Table. Listing of key features of all included studies, sorted by year of publication**

| **Authors [ref]^a^** | **Year** | **Title** | **Data collection time-frame** | **Country** | **EHRs** | **Sample size for main analysis** | **Analysis criteria** | **Percent of target population analysed** | **Percent dropped out of follow up** | **Change in renal function measure** | **Methods** | **Average follow up time for renal function** |
| --- | --- | --- | --- | --- | --- | --- | --- | --- | --- | --- | --- | --- |
| Joss N et al [69] | 2002 | Diabetic nephropathy: how effective is treatment in clinical practice? | 1989 - 1999 | UK | Clear EHRs used | 125 | ≥4 x creatinine over ≥6 months;  no death/RRT in first year | 74% | 50% | regression slope of estimated creatinine clearance | linear regression | 3 years |
| Dean BB et al [70] | 2005 | Erythropoiesis-stimulating protein therapy and the decline of renal function: a retrospective analysis of patients with chronic kidney disease | 1998-2002 | USA | Clear EHRs used | 122 | ≥4 x creatinine over ≥6 months | not available | not available | regression slope of inverse creatinine | linear regression | 1.6 years |
| Gallant JE et al [38] | 2005 | Changes in renal function associated with tenofovir disoproxil fumarate treatment, compared with nucleoside reverse-transcriptase inhibitor treatment | 2001-2004 | USA | Clear EHRs used | 658 | Unclear; assume ≥3 x creatinine over ≤1 year | not available | not available | percent change in estimated creatinine clearance from baseline | linear regression | 1 year |
| Eriksen BO et al [14] | 2006 | The progression of chronic kidney disease: a 10-year population-based study of the effects of gender and age | 1994-2003 | Norway | Clear EHRs used | 3047 | ≥2 x creatinine over ≥3 months | 100% | 34% | regression slope of eGFR (absolute scale) | linear mixed model | 3.7 years |
| Jones C et al [71] | 2006 | An evaluation of a shared primary and secondary care nephrology service for managing patients with moderate to advanced CKD | 1997-2006 | UK | Clear EHRs used | 738 | ≥6 x creatinine (3 x 5-year period pre-referral; 3 x 5-year period post-referral) | 78% | not available | regression slope of eGFR (absolute scale) | mean difference paired t-test | Not stated (max 10 years) |
| Chen SC et al [72] | 2008 | Slowing renal function decline in chronic kidney disease patients after nephrology referral | 2001-2006 | Taiwan | Clear EHRs used | 213 | ≥6 x creatinine (3 x 1-year period pre-referral; 3 x 1-year period post-referral); no AKI or dialysis ≤1 year post-referral | not available | not relevant (complete case analysis) | regression slope of eGFR (absolute scale) | generalised estimating equations | Not stated (max 2 years) |
| O’Riordan A et al [57] | 2009 | Renal biopsy in liver transplant recipients | 1996-? (likely decade 2000-2010) | UK | Clear EHRs used | 54 | Not stated | 3% | not available | binary progression to threshold eGFR | kaplan meier estimation + log-rank test | 3.2 years |
| Eriksen BO et al [17] | 2010 | Predictors of declining glomerular filtration rate in a population-based chronic kidney disease cohort | 1994-2003 | Norway | Clear EHRs used | 1224 | ≥2 x creatinine over ≥3 months | 88% | not available | regression slope of eGFR (absolute scale) | linear mixed model | 4.0 years |
| Cummings DM et al [47] | 2011 | Glycemic control patterns and kidney disease progression among primary care patients with diabetes mellitus | 1998-2008 | USA | Clear EHRs used | 791 | ≥2 x creatinine (and ≥5 x HbA1c) | 37% | not available | absolute change in eGFR from baseline | linear regression | 7.6 years |
| Abdelhafiz et al [13] | 2012 | Natural history and predictors of faster glomerular filtration rate decline in a referred population of older patients with type 2 diabetes mellitus | 1993-2010 | UK | Clear EHRs used | 100 | No creatinine criteria;  ≥5 years clinic attendance | not available | not available | regression slope of eGFR (absolute scale) | logistic regression | 14 years |
| Boudville N et al [73] | 2012 | Factors associated with Chronic Kidney Disease Progression in Australian Nephrology Practices | not stated | Australia | Clear EHRs used | 1328 | ≥2 x creatinine over ≥90 days | not available | not available | Rate of change in eGFR (not clearly defined) | linear regression | 1.5 years |
| Dreyer G et al [74] | 2013 | Progression of chronic kidney disease in a multi-ethnic community cohort of patients with diabetes mellitus | 2005-2010 | UK | Clear EHRs used | 3855 | ≥3 x creatinine over 5 years | Not available (between 60%-95% ) | 15% | regression slope of eGFR (absolute scale) | linear mixed model | 4.3 years |
| Herget-Rosenthal S et al [39] | 2013 | Progressive chronic kidney disease in primary care: modifiable risk factors predictive model | 2003-2006 | Germany | Clear EHRs used | 803 | Not clear; assume ≥2 x creatinine (2003 and 2006) | not available | not relevant (complete case analysis) | Rate of change in eGFR (not clearly defined) | logistic regression | 3 years |
| Malgor RD et al [75] | 2013 | A case-control study of intentional occlusion of accessory renal arteries during endovascular aortic aneurysm repair | 1989-2009 | USA | Not clear if EHRs used | 119 | Not stated | not available | not available | absolute change in eGFR from baseline | ANCOVA | 3.1 years |
| Brosnan EM et al [76] | 2014 | Drug-induced reduction in estimated glomerular filtration rate in patients with ALK-positive non-small cell lung cancer treated with ALK inhibitor crizotinib | 2009-2012 | USA | Clear EHRs used | 38 | Not stated | 100% | not available | regression slope of eGFR (absolute scale) | linear mixed model | 12 weeks |
| Chase HS et al [11] | 2014 | Presence of early CKD-related metabolic complications predict progression of stage 3 CKD: a case-controlled study | 2006-2012 | USA | Clear EHRs used | 481 | ≥4 x creatinine over ≥4 years | 69% | not available | regression slope of eGFR (absolute scale) | Naïve Bayes classifier; logistic regression | 6 years |
| Chen H et al [45] | 2014 | Combined application of eGFR and albuminuria for the precise diagnosis of stage 2 and 3a CKD in the elderly | 2000-2012 | China | Not clear if EHRs used | 365 | ≥3 x creatinine (and other markers) over 3 years | not available | not relevant (complete case analysis) | Rate of percentage change in eGFR (not clearly defined) | logistic regression | 3 years |
| Kose E et al [77] | 2014 | Effects on serum uric acid by difference of the renal protective effects with atorvastatin and rosuvastatin in chronic kidney disease patients | 2006-2011 | Japan | Clear EHRs used | 29 | ≥2 x creatinine over 3 months | 83% | not relevant (complete case analysis) | absolute change in eGFR from baseline | mean difference paired t-test | 3 months |
| Nderitu P et al [41] | 2014 | Analgesia dose prescribing and estimated glomerular filtration rate decline: a general practice database linkage cohort study | 2009-2010 | UK | Clear EHRs used | 4145 | ≥2 x creatinine over ≥90 days | 32% | not available | rate of change in eGFR | logistic regression | 9 months |
| Oetjens M et al [53] | 2014 | Utilization of an EMR-biorepository to identify the genetic predictors of calcineurin-inhibitor toxicity in heart transplant patients | not stated | USA | Clear EHRs used | 115 | Not stated | 91% | not available | binary progression to threshold eGFR | Cox PH regression | 8.8 years |
| Annor FB et al [18] | 2015 | Psychosocial stress and changes in estimated glomerular filtration rate among adults with diabetes mellitus | 2005-2008 | USA | Clear EHRs used | 575 | Not stated | not available | not available | regression slope of eGFR (absolute scale) | structural equation modelling | 4 years |
| Cid Ruzafa J et al [78] | 2015 | Estimated glomerular filtration rate progression in UK primary care patients with type 2 diabetes and diabetic kidney disease: a retrospective cohort study | 2006-2011 | UK | Clear EHRs used | 15692 | Not stated; assume ≥2 x creatinine over ≥1 year | 26% | not available | regression slope of eGFR (absolute scale) | linear mixed model | 3.7 years |
| Diggle PJ et al [19] | 2015 | Real-time monitoring of progression towards renal failure in primary care patients | 1997-2007+ | UK | Clear EHRs used | 22910 | ≥1 x creatinine | 100% | not available | predicted percent change in eGFR per unit time | linear mixed model | 4.5 years |
| Kaga M et al [79] | 2015 | Risk of new-onset dyslipidemia after laparoscopic adrenalectomy in patient with primary aldosteronism | 1998-2013 | Japan | Not clear if EHRs used | 57 | 2 x creatinine over 1 year | not available | not relevant (complete case analysis) | absolute change in eGFR from baseline | mean difference paired t-test | 1 year |
| Lai CL et al [80] | 2015 | Effects of atorvastatin and rosuvastatin on renal function in patients with type 2 diabetes mellitus | 2000-2010 | Taiwan | Clear EHRs used | 5569 | ≥2 x creatinine | 7% | not available | absolute change in eGFR from baseline | linear regression | 7.5 months |
| Perotte A et al [46] | 2015 | Risk prediction for chronic kidney disease progression using heterogeneous electronic health record data and time series analysis | up to 2012; approx 12 years prior follow up | USA | Clear EHRs used | 2908 | ≥2 x creatinine over ≥3 months | 100% | not available | binary progression to threshold eGFR | Kalman filter time series model; Cox PH regression | Not stated |
| Singh A et al [21] | 2015 | Incorporating temporal EHR data in predictive models for risk stratification of renal function deterioration | not stated | USA | Clear EHRs used | 6435 | ≥4 x creatinine | not available | not available | percent change in eGFR from baseline | logistic regression | Not stated |
| Vejakama P et al [25] | 2015 | Epidemiological study of chronic kidney disease progression: a large-scale population-based cohort study | 1997-2011 | Thailand | Clear EHRs used | 32106 | Not stated; assume ≥2 x creatinine over ≥3 months | not available | not available | percent change in eGFR from baseline | competing risks survival models | 4.5 years |
| Yun WS et al [81] | 2014 | Long-term follow up results of acute renal embolism after anticoagulation therapy | 2006-2012 | South Korea | Not clear if EHRs used | 31 | Not stated; assume 2 x creatinine | 66% | not available | absolute change in serum creatinine | descriptive result only; no statistical analysis | 2.6 years |
| Chakera A et al [44] | 2015 | Prognostic value of endocapillary hypercellularity in IgA nephropathy patients with no immunosuppression | not stated | UK | Clear EHRs used | 147 | Not stated | 62% | not available | Rate of change in eGFR (not clearly defined) | logistic regression | 7 years |
| Johnson F et al [43] | 2015 | The impact of acute kidney injury in diabetes mellitus | 2009-2012 | UK | Clear EHRs used | 200 | Not stated; assume ≥2 x creatinine over ≥6 months | 41% | not available | Rate of change in eGFR (not clearly defined) | difference in proportions chi-squared test | Not stated |
| Kim YG et al [82] | 2016 | Renal protective effect of DPP-4 inhibitors in type 2 diabetes mellitus patients: a cohort study | 2010-2015 | South Korea | Clear EHRs used | 414 | ≥3 x creatinine over 2 years | not available | not relevant (complete case analysis) | absolute change in eGFR from baseline | mean difference paired t-test | 2 years |
| Li XM et al [37] | 2016 | Clinicopathological characteristics and outcomes of light chain deposition disease: an analysis of 48 patients in a single Chinese center | 2004-2015 | China | Not clear if EHRs used | 44 | Not stated | 92% | not available | binary progression to threshold serum creatinine | Cox PH regression | 1.8 years |
| Mirajkar N et al [83] | 2016 | The impact of bariatric surgery on estimated glomerular filtration rate in patients with type 2 diabetes: a retrospective cohort study | 2005-2012 | UK | Clear EHRs used | 388 | ≥2 x creatinine | 49% | not available | absolute change in eGFR from baseline | simple non-parametric tests (Mann Whitney U) | 3 years |
| Koraishy FM et al [42] | 2017 | Rate of renal function decline, race and referral to nephrology in a large cohort of primary care patients | 2008-2015 | USA | Clear EHRs used | 2170 | ≥2 x creatinine over 7 years | 7% | not available | rate of change in eGFR | logistic regression | Not stated |
| Lv L et al [36] | 2017 | Persistent hematuria in patients with antineutrophil cytoplasmic antibody-associated vasculitis during clinical remission: chronic glomerular lesion or low-grade active renal vasculitis? | 1996-2016 (FU 2002-2016) | China | Not clear if EHRs used | 208 | Not stated; assume ≥2 x creatinine | 95% | not available | rate of change in eGFR | Cox PH regression | 3.1 years |
| Nishida Y et al [84] | 2017 | Comparative effect of calcium channel blockers on glomerular function in hypertensive patients with diabetes mellitus | 2004-2012 | Japan | Clear EHRs used | 1217 | Not stated | not available | not available | percent change in eGFR from baseline | linear mixed model | 1 year |
| Rincon-Choles H et al [61] | 2017 | Impact of uric acid levels on kidney disease progression | 2005-2009 | USA | Clear EHRs used | 1676 | ≥3 x creatinine; ≥1 x uric acid | 6% | not available | Binary progression (changes/threshold combination) | competing risks survival models | 2.8 years |
| Tsai CW et al [58] | 2017 | Serum Uric Acid and Progression of Kidney Disease: A Longitudinal Analysis and Mini-Review. | 2003-2011 | Taiwan | Clear EHRs used | 739 | ≥3 x creatinine over 8 years; no RRT in first 30 days | not available | not available | regression slope of eGFR (absolute scale) | linear mixed model | 4.3 years |
| Yao X et al [30] | 2017 | Renal outcomes in anticoagulated patients with atrial fibrillation | 2010-2016 | USA | Clear EHRs used | 9769 | ≥2 x creatinine | not available | not available | percent change in eGFR from baseline | Cox PH regression | 11 months |
| Beyer-Westendorf J et al [85] | 2018 | The CHA2DS2VASc score strongly correlates with glomerular filtration rate and predicts renal function decline over time in elderly patients with atrial fibrillation and chronic kidney disease | 2008-2015 | multiple european countries | Clear EHRs used | 36779 | ≥1 x creatinine | 53% Germany; not available UK | not available | regression slope of eGFR (absolute scale) | joint longitudinal survival model | 1.7 years |
| Butt AA et al [20] | 2018 | Effectiveness, treatment completion and safety of sofosbuvir/ledipasvir and paritaprevir/ritonavir/ombitasvir + dasabuvir in patients with chronic kidney disease: an ERCHVIES study | 2014-2016 | USA | Clear EHRs used | 17624 | ≥3 x creatinine over ≥6 months | 47% | 44% | absolute change in eGFR from baseline | difference in proportions chi-squared test | 12 weeks |
| Lamacchia O et al [31] | 2018 | Normoalbuminuria kidney impairment in patients with T1DM: insights from annals initiative | 2004-2011 | Italy | Clear EHRs used | 582 | ≥2 x creatinine over 4 years; ≥1 x albuminuria | 42% | not available | percent change in eGFR from baseline | logistic regression | 4 years |
| VanWagner LB et al [62] | 2018 | Cardiovascular disease outcomes related to early stage renal impairment following liver transplantation | 2002-2012 | USA | Clear EHRs used | 671 | ≥3 x creatinine over ≤1 year | not available | not available | eGFR trajectory group | trajectory clustering using latent variables | 1 year |
| Viazzi F et al [32] | 2018 | Apparent treatment resistent hypertension, blood pressure control and the progression of chronic kidney disease in patients with type 2 diabetes | 2004-2011 | Italy | Clear EHRs used | 2312 | ≥6 x creatinine over ≥4 years; Complete data for BP, eGFR and albuminuria according to treatment protocol | 33% | not relevant (complete case analysis) | percent change in eGFR from baseline | logistic regression | 4 years |
| Evans RDR et al [22] | 2018 | Clinical manifestations and long-term outcomes of IgG4-related kidney and retroperitoneal involvement in a United Kingdom IgG4-related disease cohort | 2002-2018 | UK | Not clear if EHRs used | 24 | ≥2 x creatinine | 86% | not relevant (complete case analysis) | percent change in eGFR from baseline | crude estimation / descriptive results only | 5 years |
| Horne L et al [48] | 2019 | Epidemiology and health outcomes associated with hyperkalemia in a primary care setting in England | 2009-2013 | UK | Clear EHRs used | 195178 | Not stated | not available | not available | transition to CKD stage | Crude estimation / descriptive results only | Not stated |
| Hsu TW et al [27] | 2019 | Comparison of the effects of dsnosumab and alendronate on cardiovascular and renal outcomes in osteoporotic patients | 2005-2017 | Taiwan | Clear EHRs used | 5046 | ≥2 x creatinine | not available | not available | percent change in eGFR from baseline | Cox PH regression | Max 5 years |
| Jalal K et al [15] | 2019 | Can billing codes accurately identify rapidly progressing stage 3 and stage 4 chronic kidney disease patients: a diagnostic test study | 2007-2017 | USA | Clear EHRs used | 10927 | ≥5 x creatinine over ≥3 years | 38% | not available | regression slope of eGFR (absolute scale) | linear mixed model | ≥3 years |
| Kim WJ et al [86] | 2019 | The role of a treat-to-target approach in the long-term renal outcomes of patients with gout | 2007-2018 | South Korea | Clear EHRs used | 244 | ≥2 x creatinine over ≥1 year | 93% | not available | absolute change in eGFR from baseline | logistic regression | 2 years |
| Lai YJ [24] | 2019 | Effect of weight loss on the estimated glomerular filtration rates of obese patients at risk of chronic kidney disease: the RIGOR-TMU study | 2008-2016+ | Taiwan | Clear EHRs used | 1620 | ≥3 x creatinine over ≥3 months;  Propensity score matched only | 37% | not available | percent change in eGFR from baseline | Cox PH regression | Max 1 year |
| Leither MD et al [59] | 2019 | The impact of outpatient acute kidney injury on mortality and chronic kidney disease: a retrospective cohort study | not stated | USA | Clear EHRs used | 196209 | Not clear; assume ≥1 x creatinine | 51% | not available | Binary progression (eGFR changes/threshold combination) | Cox PH regression | 5.3 years |
| Liu D et al [60] | 2019 | Serum immunoglobin G provides early risk prediction in immunoglobin A nephropathy | 2009-2014 | China | Not clear if EHRs used | 455 | Not clear but ≥2 years; 1 x serum IgG | 73% | not available | Binary progression (eGFR changes/threshold combination) | Cox PH regression | 3.7 years |
| Morales-Alvarez MC et al [40] | 2019 | Renal function decline in latinos with type 2 diabetes | 2002-2015 | USA | Clear EHRs used | 594 | ≥2 x creatinine | 65% | not available | rate of change in eGFR | difference in means t-test | Not stated |
| O’Neill RA et al [87] | 2019 | Evaluation of long-term intravitreal antivascular endothelial growth factor injections on renal function in patients with and without diabetic kidney disease | 2012-2018 | UK | Clear EHRs used | 85 | Not stated | not available | not available | regression slope of eGFR (absolute scale) | linear regression | 2.6 years |
| Park JM et al [88] | 2018 | Oncological and functional outcomes of laparoscopic radiofrequency ablation and partial nephrectomy for T1a renal masses: a retrospecive single-center 60 month follow up cohort study | 2005-2014 | South Korea | Not clear if EHRs used | 115 | Not stated but ≥2 years | not available | not available | absolute change in eGFR from baseline | difference in means t-test | 5.3 years |
| Posch F et al [89] | 2019 | Longitudinal kidney function trajectories predict major bleeding, hospitalisation and death in patients with atrial fibrillation and chronic kidney disease | 2009-2015 | UK | Clear EHRs used | 18240 | Not clear but ≥6 months; assume ≥1 x creatinine | 88% | not available | rate of change in eGFR | joint longitudinal survival model | 1.9 years |
| Posch F et al [26] | 2019 | Exposure to vitamin k antagonists and kidney function decline in patients with atrial fibrillation and chronic kidney disease | 2009-2015 | Germany | Clear EHRs used | 14432 | ≥1 x creatinine; ≥1 x CHA2DS2VASc score | 39% | not available | regression slope of eGFR (absolute scale and percent scale) | linear mixed model | 1.4 years |
| Spanopoulos D et al [90] | 2019 | Temporal variation of renal function in people with type 2 diabetes mellitus: a retrospective UK clinical practice research datalink cohort study | 2009-2016 | UK | Clear EHRs used | 7766 | ≥6 x creatinine over 5 years | 17% | not available | transition to CKD stages | descriptive result only; no statistical analysis | 5 years |
| Wang Y et al [12] | 2019 | Implications of a family history of diabetes and rapid eGFR decline in patients with type 2 diabetes and biopsy-proven diabetic kidney disease | 2007-2017 | China | Clear EHRs used | 128 | ≥3 x creatinine over ≥1 year | not available | not available | regression slope of eGFR (absolute scale) | logistic regression | 2 years |
| Yoo H et al [34] | 2019 | Effects of sarpogrelate on microvascular complications with type 2 diabetes | 2010-2015 | South Korea | Clear EHRs used | 478 | Not clear; assume ≥2 x creatinine; Propensity score matched only | 9% | not available | percent change in eGFR from baseline | kaplan meier estimation + log-rank test | 5.7 years |
| Zhao J et al [91] | 2019 | Predicting outcomes of chronic kidney disease from EMR data based on random forest regression | 2009-2017 | USA | Clear EHRs used | 61740 | ≥3 x creatinine over 7 years | 51% | not available | eGFR prediction at multiple time points | random forest regression | Not stated |
| Lee JS et al [92] | 2020 | Recovery of renal function in patients with lupus nephritis and reduced renal function: the beneficial effect of hydroxychloroquine | 1995-2018 | South Korea | Clear EHRs used | 90 | ≥2 x creatinine over 6 months | 100% | not relevant (complete case analysis) | binary progression to threshold eGFR | logistic regression | 6 months |
| Nakamura A et al [93] | 2020 | Impact of sodium-glucose cotransporter 2 inhibitors on renal function in participants with type 2 diabetes and chronic kidney disease with normoalbuminuria | not stated | Japan | Not clear if EHRs used | 87 | ≥2 x creatinine over 2 years | not available | not relevant (complete case analysis) | percent change in eGFR from baseline | ANOVA | 2 years |
| Sise ME et al [94] | 2019 | Direct-acting antiviral therapy slows kidney function decline in patients with Hepatitis C virus infection and chronic kidney disease | 2013-2017 | USA | Clear EHRs used | 1178 | ≥4 x creatinine over ≤6 years | 60% | not available | regression slope of eGFR (absolute scale) | generalised estimating equations | 1.6 years (post-therapy) |
| Weldegiorgis M et al [55] | 2020 | Socioeconomic disadvantage and the risk of advanced chronic kidney disease: results from a cohort study with 1.4 million participants | 2020-2014+ | UK | Clear EHRs used | 1,397,573 | ≥2 years “data” before baseline; ≥3 years follow-up; no specific creatinine requirement | not available | not available | Binary progression to threshold eGFR | Cox PH regression | 7.5 years |
| Cabrera CS et al [16] | 2020 | Impact of CKD Progression on Cardiovascular Disease Risk in a Contemporary UK Cohort of Individuals With Diabetes | 2005-2015 | UK | Clear EHRs used | 3,022 | ≥2 x creatinine at baseline; ≥1 x creatinine follow-up | 57% | Not available | regression slope of eGFR (absolute scale) | Cox PH regression | 4.3 years |
| Cleary F et al [95] | 2020 | Feasibility of evaluation of the natural history of kidney disease in the general population using electronic healthcare records | 2008-2016 | UK | Clear EHRs used | 1,597,629 | ≥3 x creatinine | 25% | 2.4% | regression slope of eGFR (absolute scale) | Linear regression | 5.7 years |
| Faraj KS et al [96] | 2020 | The effect of urinary diversion on long-term kidney function after cystectomy | 2007-2018+ | USA | Clear EHRs used | 563 | ≥1 x creatinine follow-up; unclear if baseline requirements | 98% | Not available | regression slope of eGFR (absolute scale) | Linear mixed model | 3.9 years |
| Inaguma D et al [28] | 2020 | Increasing tendency of urine protein is a risk factor for rapid eGFR decline in patients with CKD: A machine learning-based prediction model by using a big database | 2004-2019 | Japan | Clear EHRs used | 9,911 | Unclear | Not available | Not relevant (complete case analysis) | Rate of percent change in eGFR, not clearly defined | Logistic regression; Random forest regression | Not stated |
| Nichols GA et al [50] | 2020 | Kidney disease progression and all-cause mortality across estimated glomerular filtration rate and albuminuria categories among patients with vs. Without type 2 diabetes | 2006-2016 | USA | Clear EHRs used | Approx. 36,727 | ≥1 x creatinine baseline; ≥1 x creatinine follow-up; ≥1 x proteinuria/ ACR at baseline | 47% | Not available | Transition between CKD stages | Life-table analysis | 5 years |
| Peng YL et al [29] | 2020 | Comparison of uric acid reduction and renal outcomes of febuxostat vs allopurinol in patients with chronic kidney disease | 2010-2015 | Taiwan | Clear EHRs used | 1,050 | ≥2 x creatinine baseline; Creatinine, SUA follow-up (unclear) | Not available | Not available | regression slope of eGFR (absolute scale) | Linear mixed model | 1.5 years |
| Rej S et al [33] | 2020 | Association of Lithium Use and a Higher Serum Concentration of Lithium With the Risk of Declining Renal Function in Older Adults: A Population-Based Cohort Study | 2007-2015 | Canada | Clear EHRs used | 6,226 | ≥1 x creatinine; ≥1 x lithium | Not available | Not available | percent change in eGFR from baseline | Cox PH regression | 3.1 years |
| Jackevicius CA et al [23] | 2021 | Bleeding Risk of Direct Oral Anticoagulants in Patients With Heart Failure And Atrial Fibrillation. Circulation-Cardiovascular Quality and Outcomes | 2010-2018 | USA | Clear EHRs used | 49,458 | Unclear | 92% | Not available | Rate of percent change in eGFR, not clearly defined | Cox PH regression | 1.4 years |
| Neuen BL et al [54] | 2021 | Changes in GFR and Albuminuria in Routine Clinical Practice and the Risk of Kidney Disease Progression | 2000-2015 | UK | Clear EHRs used | 91,319 | ≥2 x creatinine; ≥2 x UACR | 1% | Not available | Percent change in eGFR between measures | Cox PH regression | 2.9 years |
| Niu SF et al [56] | 2021 | Early Chronic Kidney Disease Care Programme delays kidney function deterioration in patients with stage I-IIIa chronic kidney disease: an observational cohort study in Taiwan | 2012-2017+ | Taiwan | Clear EHRs used | 3,114 | Unclear; ≥2 x medical visits; possibly ≥1 x creatinine | Not available | Not available | Binary progression to threshold eGFR | Cox PH regression | 3.0 years |
| Robinson DE et al [49] | 2021 | Safety of Oral Bisphosphonates in Moderate-to-Severe Chronic Kidney Disease: A Binational Cohort Analysis. Journal of Bone and Mineral Research | 1997-2016 | Multiple European countries | Clear EHRs used | 19,324 | ≥2 x creatinine at baseline; ≥1 x creatinine follow-up | Not available | Not available | Transition between CKD stages | Competing risks survival models | 3.7 years |
| Tangri N et al [35] | 2021 | Metabolic acidosis is associated with increased risk of adverse kidney outcomes and mortality in patients with non-dialysis dependent chronic kidney disease: an observational cohort study | 2007-2017 | USA | Clear EHRs used | 32,007 | ≥3 x creatinine; ≥3 x serum bicarbonate | Not available | Not available | percent change in eGFR from baseline | Cox PH regression | 3.9 years |
| Vesga JI et al [52] | 2021 | Chronic kidney disease progression and transition probabilities in a large preventive cohort in colombia | 2009-2018 | Colombia | Clear EHRs used | 2,752 | ≥2 x creatinine | 90% | 65% | Transition between CKD stages | Crude estimation | Not stated |
| Yanagawa et al [51] | 2021 | Retrospective study of factors associated with progression and remission/regression of diabetic kidney disease-hypomagnesemia was associated with progression and elevated serum alanine aminotransferase levels were associated with remission or regression | 2003-2019 | Japan | Clear EHRs used | 681 | Not stated | 99% | Not relevant (complete case analysis) | Transition between CKD stages | Cox PH regression | 6.2 years |

^a^[ref] refers to reference number in supplementary listing of reviewed studies (which also match those listed in manuscript Table 4)
