# Supplementary material for: A systematic review of statistical methodology used to evaluate progression of chronic kidney disease using electronic healthcare records
Source: PLoS One. 2022 Jul 29;17(7):e0264167. doi: 10.1371/journal.pone.0264167 (PMC9337679; doi:10.1371/journal.pone.0264167)
Supplement: Attachment — Submitted filename: Response to Reviewers.docx [file pone.0264167.s009.docx]

Faye Cleary
London School of Hygiene and Tropical Medicine
Keppel Street
London
WC1E 7HT
faye.cleary@lshtm.ac.uk

Stanislaw Stepkowski

Academic Editor

PLOS ONE

26^th^ October 2021

Dear Dr. Stepkowski,

Thank you very much for your consideration of the manuscript “A systematic review of statistical methodology used to evaluate progression of chronic kidney disease using electronic healthcare records” for publication in PLOS ONE, and for the valuable feedback provided by reviewers. I apologise for the delays in re-submitting this work, as I have worked to update the review among other competing priorities. We have now updated the manuscript to address the reviewer comments, which includes extending the search dates of the review to August 2021 and other clarifications of the text to support clarity and readability of the review. I respond to specific reviewer comments individually below, in bold text.

*Reviewer #1:
The systematic analysis by Faye Cleary et al. is useful in terms of identifying the opportunities coming from increasing availability of electronic health records, as well as pointing to common mistakes and challenges associated with their intrinsic nature. In this regard, the authors did a good job summarizing the methodology across the 65 included studies. It would be nice to note that retrospective studies using traditional paper records will suffer from the same problems as those using electronic health records: incomplete records, variation in logging practices, addressing AKI when modeling CKD progression, loss to follow-up and competing risks. I don't have other concerns about this work.*
**We are pleased that the reviewer sees the value and quality of our work. In response to reviewer suggestions, we have updated text in the discussion to note that traditional paper records will suffer from the same problems as those using electronic healthcare records.**

*Comments Reviewer #2:
A well written systematic review with proper design, presentation of result, and discussion.*
**We are pleased that the reviewer believes we have conducted a well-designed and presented review of the literature.**
*I have few comments:
-The search needs to be updated beyond 7th May 2020. Multiple studies were published after this date.*
**We have updated the search dates to include studies available in the 4 databases covered by the review as of August 2021, allowing us to capture more recently published studies.** *-Clarify the study methodology: Sample size (before and after exclusions for reasons of data completeness);*
**We have clarified in the methods text that data completeness inclusion criteria refer to the specific study inclusion criteria applied prior to main analyses being performed that aimed to restrict analyses to only those patients with sufficient data completeness to be deemed suitable for analysis, with such criteria expected to vary between studies. The explanation of the calculation for “percent of target population analysed” also shows readers how we used sample size data before and after data completeness inclusion criteria were applied to uncover the extent to which patients were excluded from analysis purely due to failure to meet a study’s data completeness requirements.**
*-The authors stated that “It is likely that if research is missing from review, then data quality issues in missing studies are likely to be of a similar quality or worse quality than studies reviewed”.  Only studies reported in English language were included in this review. Therefore,  it is unfair to label studies in non-English language as inferior in quality.*
**Our comment that “With peer-reviewed literature expected to go through certain research quality checks, it is likely that if research is missing from review, then data quality issues in missing studies are likely to be of a similar quality or worse quality than studies reviewed” was intended to convey that studies missing from review due to not being peer-reviewed are likely to be of similar or worse quality as/than those peer-reviewed, due to the quality checks that peer-reviewed studies go through. It was not intended to say anything about studies published in non-English languages. We have clarified in the methods text that we anticipate that studies published in both English and non-English languages are likely to be of similar quality.** *-What is the outcome for Cox proportional hazards regression? I assume time to event !!*
**I’m not 100% sure where exactly in the manuscript the reviewer is referring to in this comment, but I imagine it may be results Tables 2 and 4. I would like to clarify what is reported and what is not. Due to the anticipated variation in how researchers define progression of kidney disease over time and the challenges this may pose in clinical interpretability of findings of research studies, a key aim of our review was to summarise how researchers measured changes in renal function over time. We also reported methods for analysis (which include as the reviewer states Cox proportional hazards regression models with such models using as outcome time to some event). In our reporting of study methodology (Table 2), we summarise “Measure of change in renal function over time”. As an example, an event of a 30% decline in eGFR between measures would be reported as “Raw percent change in eGFR between measures” (as this captures how changes over time were measured) and we do not specifically state whether this was analysed as time to event or as a binary outcome but we do report the method of analysis (“Statistical model used”), for example Cox proportional hazards regression. Table 4 further clarifies precise measures of changes in renal function over time for each individual study (e.g. percent loss in eGFR between measures >30%) alongside the methods used (e.g. Cox PH regression). Although we do not specifically state what the outcome is (e.g. time to percent loss in eGFR between measures >30%), this is inferred. We have added a comment to Table 4 to clarify this.**

In response to additional journal requirements, I have uploaded my raw data spreadsheet alongside my updated manuscript and will also make this available via LSHTM Data Compass repository. The manuscript reference list has not been updated. However, the list of reviewed articles has been updated and will be uploaded online as supplementary materials.

Thank you again for your consideration of this manuscript.

Sincerely,

Faye Cleary

Department of Non-Communicable Disease Epidemiology,

London School of Hygiene and Tropical Medicine
